# Twenty-four-hour movement patterns relate with cardiometabolic health in adults with obesity

**DOI:** 10.1007/s11332-026-01863-x

**Published:** 2026-08-06

**Authors:** Kevin Y. Gendi, Emily M. Heiston, Daniel J. Battillo, Haiqun Lin, Steven K. Malin

**Affiliations:** 1 Department of Kinesiology and Health, Rutgers University, 70 Lipman Dr, Loree Gymnasium, New Brunswick, NJ 08901 USA; 2 Division of Nursing, Rutgers University, Newark, NJ USA; 3 Division of Endocrinology, Metabolism and Nutrition, Rutgers University, New Brunswick, NJ USA; 4 New Jersey Institute for Food, Nutrition and Health, Rutgers University, New Brunswick, NJ USA; 5 Institute of Translational Medicine and Science, Rutgers University, New Brunswick, NJ USA

**Keywords:** Physical activity, Sleep, Sedentary behavior, Type 2 diabetes, Cardiovascular disease, Insulin resistance

## Abstract

**Aim:**

Low moderate-to-vigorous physical activity (MVPA), high sedentary behavior (SB), and inadequate sleep are individually associated with type 2 diabetes (T2D) risk. However, the aggregate impact of these movement patterns remains unclear.

**Purpose:**

Assess whether being aligned with 24-h movement guidelines of MVPA, SB, and sleep relates to cardiometabolic health, and compare this with the American Heart Association’s Life’s Essential 8.

**Methods:**

In a cross-sectional study, adults with obesity [*n* = 56 (39 F); 55.6 ± 1.1 y; 34.5 ± 0.7 kg/m^2^] with low 24-h movement *z*-scores [“aligned”; *n* = 28 (21 F)] were compared to those with high *z*-scores [“misaligned” *n* = 28 (18 F)]. MVPA and SB (accelerometer) and sleep (Pittsburgh Sleep Quality Index) were assessed to calculate *z* scores. LE8 scores were calculated according to AHA guidelines. A 120-min hyperinsulinemic-euglycemic clamp (40 mU/m^2^/min, 90 mg/dl) with indirect calorimetry was used to determine metabolic insulin sensitivity [glucose infusion rate (GIR)] and substrate oxidation. Pulse wave velocity and augmentation index (AIx75) were assessed at 0 and 120 min of the clamp to assess arterial stiffness. Fitness (*V*O_2max_) and body fat (DXA) were also assessed.

**Results:**

Despite no differences in age, body fat, or *V*O_2max_, the misaligned group had higher fasting DBP, MAP, and AIx75 (*P* < 0.05), lower GIR (*P* = 0.07), and lower FFA suppression (*P* = 0.01). While both the *z*-score and LE8 related to blood pressure, only the *z*-score correlated with AIx75 and FFA suppression.

**Conclusion:**

Aligning with 24-h movement patterns related to better cardiometabolic health in people with obesity.

**Clinical trials registration:**

NCT03355469.

## Introduction

Approximately 40% of adults in the U.S. have obesity [1], and an estimated 50% of U.S. adults do not meet MVPA requirements [2]. In fact, nearly 25% of these adults exhibit excessive SB [3] and 35% get inadequate sleep time [4]. This is problematic as each of these lifestyle behaviors is not only linked to obesity, but also associated with type 2 diabetes (T2D) and cardiovascular disease (CVD) risk [5]. Studies have increasingly pointed to the individual health benefits of meeting MVPA requirements [6], decreasing SB [7], and achieving adequate sleep [8]. However, little is known about how the combination of these movement patterns work together to affect health [5].

The Canadian Society for Exercise Physiology (CSEP) recently published 24-h movement guidelines to optimize health [9]. These guidelines consider the integration of MVPA, SB, and sleep within a 24-h period. Because the proportion of time spent in each movement pattern is collinear, an increase in time spent in one movement pattern causes a decrease in time spent in another [5]. However, no clear assessment approach is available to measure this composite of 24-h movement patterns and understand its relation to insulin resistance, obesity, or CVD risk. Thus, the purpose of this study was to identify if a 24-h movement pattern z-score of MVPA, SB, and sleep associates with cardiometabolic health in adults with obesity. Further, the American Heart Association (AHA) recently released an updated version of their construct of cardiovascular health, the Life’s Essential 8 (LE8), which uses physical activity (PA) and sleep, among other clinical risk factors, to create a score which may predict cardiovascular health [10]. Thus, we secondarily sought to compare the 24-h movement z-score to the LE8 score to understand its utility in relating to CVD risk.

## Methods

### Study design and participants

Participants (*n* = 56, Table 1) with obesity baseline data who were part of a larger randomized controlled trial (#NCT03355469) were used in this cross-sectional study. Participants were recruited via social media, electronic medical records, and/or newspaper flyers from the local communities. Individuals were included if between the ages of 40 and 70 year, non-smoking, physically inactive (exercise < 150 min/week), weight stable (< 2 kg weight change during last 3 months), not taking medications known to affect glucose metabolism (e.g., biguanides, insulin, etc.), and free of chronic disease (e.g., renal, hepatic, etc.). Female participants were asked to indicate menses status. Participants underwent ECG testing before and during exercise to assess cardiac function. Biochemical analysis as well as a physical examination were also done to confirm eligibility. Biochemical analysis included a fasted lipid profile, comprehensive metabolic panel, and HbA1c blood test to characterize cardiometabolic risk. Blood pressure (BP) was also measured at time of the physical examination using an automatic BP cuff on the upper arm. Heart rate was recorded too, using pulse oximetry. Mean arterial pressure (MAP) was calculated as MAP = (2/3xDBP) + (1/3xSBP). Participants provided written and verbal informed consent prior to participation. 

**Table 1:** Demographics and 24-h movement patterns

	Aligned 24-h movement pattern	Misaligned 24-h movement pattern	*P *value
*N* (female)	28 (21)	28 (18)	
Post-menopause	14	13	
Age (years)	55.36 ± 1.58	55.82 ± 1.58	0.837
Weight (kg)^a^	93.16 ± 3.21	103.74 ± 3.84	0.042
Waist circumference (cm)^a^	106.31 ± 2.43	110.14 ± 2.54	0.284
Body Mass Index (kg/m^2^)^b^	31.95(29.60–37.43)	36.05(31.35–39.95)	*U* = 285.0, *P* = 0.079
Lean mass (kg)^a^	50.69 ± 2.29	53.44 ± 2.24	0.352
Body fat (%)	42.94 ± 1.27	45.01 ± 1.28	0.258
Visceral fat mass (kg)^a^	1.30 ± 0.15	1.73 ± 0.19	0.056
*V*O_2max_ (ml/kg/min)	23.03 ± 0.78	23.76 ± 0.87	0.536
HbA1c (%)^b^	5.60(5.43–5.90)	5.60(5.43–5.98)	U = 374.5, *P* = 0.773
Triglycerides (mg/dl)^a^	132.93 ± 10.43	137.93 ± 14.10	0.900
HDL (mg/dl)^a^	47.36 ± 2.27	51.43 ± 2.20	0.185
Cholesterol (mg/dl)	184.18 ± 8.28	201.36 ± 9.10	0.168
LDL (mg/dl)	113.82 ± 7.07	123.54 ± 8.40	0.380
SBP (mmHg)	128.19 ± 2.56	134.17 ± 2.87	0.126
DBP (mmHg)	77.24 ± 1.67	83.15 ± 1.58	0.013
MAP (mmHg)	94.22 ± 1.79	100.15 ± 1.92	0.028
DASH Score (a.u.)	2.53 ± 0.34	2.06 ± 0.22	0.257
LE8 score (a.u.)	65.07 ± 1.61	56.99 ± 2.37	0.009
24-h *z*-score (a.u.)	3.64 ± 0.25	6.84 ± 0.23	< 0.001
MVPA *z*-score (a.u.)	− 1.30 ± 0.19	− 0.12 ± 0.10	< 0.001
Sleep *z*-score (a.u.)	− 0.13 ± 0.17	0.74 ± 0.18	0.001
Sedentary *z*-score (a.u.)	5.07 ± 0.19	6.22 ± 0.13	< 0.001

*V*O_2max_ cardiorespiratory fitness, *HbA1c* hemoglobin A1c, *HDL* high-density lipoprotein, *LDL* low-density lipoprotein, *SBP* systolic blood pressure, *DBP* diastolic blood pressure, *MAP* mean arterial pressure, *DASH* score Dietary Approaches to Stop Hypertension, *LE8* Life’s Essential 8 score, *MVPA* moderate-to-vigorous physical activity

^a^Data are log-transformed for analysis, but raw data are presented for interpretation. Normally distributed data are mean ± SEM

^b^Non-normally distributed data are median (interquartile range)

### Body composition and fitness

Body weight was recorded using a digital scale, and height was recorded with a stadiometer to calculate body mass index (BMI). Body fat, lean mass, and visceral fat mass were assessed via dual-energy X-ray absorptiometry [11]. Cardiorespiratory fitness (*V*O_2max_) was tested via a treadmill with indirect calorimetry as previously described [11]. Resting metabolic rate (RMR) was determined after an overnight fast using indirect calorimetry for approximately 20 min with a ventilated hood (Carefusion, *V*_max_ CART, Yorba Linda, CA, USA or Cosmed, Quark RMR, Concord, CA, USA). Indirect calorimetry was calibrated per manufacturer recommendations prior to each data collection. Flow rates for the canopy system were adjusted accordingly to ensure CO_2_ concentrations were within manufacturer range of approximately 1%. Data were then obtained during the last 5 min to ensure that the coefficient of variation was < 10% for each *V*O_2_ and *V*CO_2_ to consider measurements valid. The last 5 min were then averaged to determine RMR, and an activity factor of 1.2 was applied to estimate daily caloric needs prior to the clamp [11].

### Clamp procedure

Participants arrived at the Clinical Research Center following an approximate 10-h overnight fast and abstaining for the last 24 h without caffeine, alcohol, or medication. Participants were instructed to only consume water and the provided mixed-meal diet (∼55% CHO, 30% fat, and 15% protein) the day prior. A catheter was placed in both the antecubital and forearm or dorsal hand veins for infusion and blood drawing, respectively. A primed (250 mU/m/min) constant infusion (40 mU/m/min) of insulin was delivered for 120 min [11]. Glucose infusion rates (GIR) were adjusted every 5 min to maintain plasma glucose at 90 mg/dL, which was analyzed using the glucose-oxidase method (YSI Instruments 2300, Yellow Springs, OH, USA). Plasma insulin and free fatty acids (FFA) were measured at 0, 90, 105, and 120 min of the clamp. Non-fasting values were averaged to assess steady-state. Insulin and FFA samples were centrifuged at 4 °C and 1500*g* and stored at − 80 °C until later analysis via ELISAs and colorimetric assays (ALPCO, Salem, NH, USA and Fuji Wako, Richmond, VA, USA). GIR was used to determine metabolic insulin sensitivity. Indirect calorimetry was performed before infusion and during steady-state to estimate carbohydrate (CHOox) and fat (FOX) oxidation. Nonoxidative glucose disposal (NOGD) was calculated as GIR minus total steady-state CHOox to estimate glucose storage. FFA suppression was calculated as [1 − (FFA_Clamp_/FFA_fasting_)] × 100 as an index for adipose tissue insulin sensitivity.

### Arterial stiffness

Aortic waveforms were measured at 0 and 120 min of the clamp with applanation tonometry (SphygmoCor XCEL system, AtCor Medical, Itasca, IL, USA), as described before [12]. Augmentation pressure (AP) and augmentation index corrected for a standardized heart rate of 75 beats per minute (AIx75) were obtained. Then participants remained in the supine position for assessment of arterial stiffness via carotid-femoral pulse wave velocity (PWV).

### Dietary analysis

Participants were provided with dietary logs and instructed to record their food intake. Diet logs were analyzed via the Food Processor (ESHA Research, Salem, OR, USA) and dietary patterns were subsequently calculated using the Mellen Dietary Approaches to Stop Hypertension (DASH) score [13].

### 24-h movement pattern z-score

Participants wore an accelerometer (wGT3X-BT, ActiGraph, Pensacola, FL, USA) around their waist for 7-d and were instructed to maintain typical physical activity levels. Time spent in MVPA and SB was calculated using ActiLife Data Analysis Software (v.6.13.6) and averaged over the 7-d to quantify daily movement patterns. Average daily sleep time over a one-month timespan was assessed via the Pittsburgh Sleep Quality Index (PSQI). Using CSEP guidelines, aligned movement patterns were defined as: ≥ 150 min/week of MVPA, ≥ 7 h/d of sleep, and ≤ 8 h/day (33% of the day) of SB. The calculation for the 24-h movement z-score was [(150 − MVPA)/174.9] + [(7 − Sleep Duration)/1.02] + [(SB − 0.33)/0.08]. A low *z*-score was considered “aligned” with guidelines versus high z-scores.

### AHA LE8 score

The LE8 score was calculated averaging the score of the 8 components according to the AHA guidelines [14]. It should be noted that we used the Mellen DASH diet index and it was scored as: 0= 0–2.99, 50 = 3–5.99, and 100 = 6–9. In this calculation, higher LE8 scores are considered more aligned with guidelines.

### Statistical analysis

Data were analyzed using SPSS (IBM, V. 28.0, Armonk, NY, USA). To understand the clinical relevance of the 24-h movement *z*-scores, we separated participants into an aligned 24-h movement pattern or misaligned 24-h movement pattern group based on the 50th percentile. Normally distributed data were analyzed by an unpaired, two-tailed *t* test. Non-normally distributed data were analyzed using the Mann–Whitney *U* test. Pearson or Spearman correlation tests were used as appropriate. Mediation analysis was conducted via SAS [15] to assess whether the 24-h movement *z*-score mediated the separate effect of baseline measurement fasting AIx75 and AP on the outcome of steady-state FFA. Using the potential outcome framework [16], two regression models were used; in the first regression model, the 24-h movement z-score was regressed on one of the measurements, and in the second regression model, FFA was regressed on the measurement and the 24-h movement *z*-score. Both regression models were adjusted for age and sex. Significance was accepted as *P* ≤ 0.05.

## Results

### Demographics

Individuals with an aligned 24-h movement pattern had better MVPA, sleep, SB, and LE8 scores (all *P* < 0.05, Table 1). While those with aligned 24-h movement patterns had lower body weight (*P* = 0.042), there were no differences in waist circumference, body composition, *V*O_2max_ or blood lipids (Table 1) between groups. However, more aligned LE8 scores correlated with lower weight, waist circumference, BMI, and visceral fat mass (Table 2).

### Blood pressure and arterial stiffness

Although SBP was not different between aligned and misaligned 24-h movement z-score groups, we did observe that fasting DBP, MAP, and AIx75 were lower in the aligned 24-h movement pattern group (all *P* < 0.05, Tables 1 and 3). More aligned 24-h movement z-scores are also associated with lower DBP, MAP, and AIx75 (Table 3). Similarly, a more aligned LE8 score also correlated with lower DBP and MAP, in addition to SBP. However, there were no differences in PWV between 24-h movement pattern groups, nor were correlations observed with respective scores (Table 4).

**Table 2:** Body composition and fitness correlation with 24-h movement pattern z-score and LE8 score

	24-h movement z-score	LE8 Score
	*R*-value, *P*-value	*R*-value, *P*-value
Weight (kg)^a^	0.197, 0.146	– 0.457, *P* = 0.007
Waist circumference (cm)^a^	0.081, 0.553	– 0.456, *P* = 0.008
Body Mass Index (kg/m^2^)^b^	0.209, 0.122	– 0.493, *P* = 0.004
Lean mass (kg)^a^	0.046, 0.756	– 0.339, *P* = 0.067
Body fat (%)	0.272, 0.059	– 0.229, *P* = 0.214
Visceral fat mass (kg)^a^	0.224, 0.121	– 0.475, *P* = 0.007
*V*O_2max_ (ml/kg/min)	0.003, 0.981	0.181, *P* = 0.313
SBP (mmHg)	0.124, 0.362	– 0.444, *P* = 0.010
DBP (mmHg)	0.355, 0.007	– 0.580, *P* < 0.001
MAP (mmHg)	0.269, 0.045	– 0.554, *P* < 0.001

*VO*_*2*_*max* cardiorespiratory fitness, *SBP* systolic blood pressure, *DBP* diastolic blood pressure, *MAP* mean arterial pressure

^a^Data log-transformed for analysis

^b^Spearman correlation was used

### Metabolic insulin sensitivity

Metabolic insulin sensitivity and NOGD (Table 5) tended to be higher in those with aligned versus misaligned 24-h movement patterns. There were no correlations between 24-h movement *z*-scores and substrate oxidation, although a more aligned LE8 score related to fasting FOX (Table 6). Nonetheless, the aligned 24-h movement pattern group had higher FFA suppression (Table 5), and FFA suppression did not relate to the LE8 score (Table 6).

**Table 3:** Arterial health and 24-h movement patterns

	Aligned 24-h movement pattern	Misaligned 24-h movement pattern	*P* value
*Fasting*			
PWV (m/s)	7.69 ± 0.37	7.43 ± 0.34	0.615
AIx75 (mmHg)^a^	28.56 ± 1.87	34.11 ± 1.94	0.033
AP (mmHg)^a^	15.26 ± 1.03	18.30 ± 1.23	0.068
Heart Rate (bpm)	63.04 ± 1.42	63.89 ± 1.86	0.717
*Insulin-stimulated*			
PWV (m/s)	7.68 ± 0.43	7.74 ± 0.32	0.917
AIx75 (mmHg)	24.00 ± 2.11	26.88 ± 2.28	0.361
AP (mmHg)	12.41 ± 1.24	13.81 ± 1.14	0.411
Heart Rate (bpm)	65.00 ± 1.52	65.50 ± 2.17	0.852

*PWV* pulse wave velocity, *AIx75* augmentation index adjusted to 75 bpm heart rate, *AP* augmentation pressure

^a^Data are log-transformed for analysis, but raw data are presented for interpretation. Normally distributed data are mean ± SEM

**Table 4:** Arterial health correlation with 24-h movement pattern z-score and LE8 score

	24-h movement pattern *z*-score	LE8 Score
	*R*-value, *P*-value	*R*-value, *P*-value
*Fasting*		
PWV (m/s)	– 0.258, 0.095	– 0.063, 0.758
AIx75 (%)^a^	0.423, 0.001	– 0.233, 0.199
AP (mmHg)^a^	0.388, 0.004	– 0.217, 0.232
Heart rate (bpm)	– 0.046, 0.740	– 0.081, 0.660
*Insulin-stimulated*		
PWV (m/s)	– 0.252, 0.157	0.130, 0.597
AIx75 (%)	0.372, 0.021	0.125, 0.580
AP (mmHg)	0.394, 0.014	– 0.167, 0.456
Heart Rate (bpm)	– 0.079, 0.638	0.279, 0.208

*PWV* pulse wave velocity, *AIx75* augmentation index adjusted to 75 bpm heart rate, *AP* augmentation pressure

^a^Data log-transformed for analysis

**Table 5:** Metabolic outcomes and 24-h movement patterns

	Aligned 24-h movement pattern	Misaligned 24-h movement pattern	*P* value
*Fasting*			
Glucose (mg/dl)^a^	99.10 ± 2.18	95.33 ± 2.81	0.227
Insulin (μU/ml)^a^	11.18 ± 1.61	12.09 ± 1.49	0.432
Lactate (mg/dl)	0.89 ± 0.05	0.86 ± 0.03	0.640
FFA (mEq/L)	0.68 ± 0.05	0.70 ± 0.03	0.744
CHOox (mg/kg/min)^b^	1.11 (0.68–1.71)	1.29 (0.55–2.28)	*U* = 325.0, *P* = 0.819
FOX (mg/kg/min)^b^	0.57 (0.37–0.89)	0.60 (0.30–0.96)	*U* = 319.0, *P* = 0.735
*Insulin-stimulated*			
Glucose (mg/dl)^b^	99.65(89.90–103.00)	93.98 (84.30–102.50)	*U* = 304.0, *P* = 0.692
Insulin _Steady-State_ (μU/ml)	80.29 ± 2.98	75.39 ± 4.69	0.382
Lactate _Steady-State_ (mg/dl)^a^	0.90 ± 0.04	0.97 ± 0.05	0.246
FFA _Steady-State_ (mEq/L)^b^	0.14 (0.11–0.19)	0.20 (0.14–0.26)	*U* = 168.5, *P* = 0.003
GIR (mg/kg/min)^b^	2.90 (1.77–4.12)	2.12 (1.69–2.48)	*U* = 260.0, *P* = 0.072
NOGD (mg/kg/min)^b^	2.81 (2.14–3.75)	2.09 (1.34–2.96)	*U* = 222.0, *P* = 0.052
CHOox (mg/kg/min)^b^	1.23 (0.92–1.98)	1.30 (0.81–2.10)	*U* = 304.0, *P* = 0.692
FOX (mg/kg/min)	0.53 ± 0.10	0.60 ± 0.10	0.634
FFA Suppression^b^	79.74 (70.85–82.95)	74.21 (53.52–78.48)	*U* = 178.0, *P* = 0.010

*FFA* free fatty acids, *CHOox* carbohydrate oxidation, *FOX* fat oxidation, *GIR* glucose infusion rate, *NOGD* non-oxidative glucose disposal

^a^Data log-transformed for analysis, but raw data are presented for interpretation. Normally distributed data are mean ± SEM

^b^Non-normally distributed data are median (interquartile range)

**Table 6:** Metabolic outcomes correlation with 24-h movement pattern *z*-score and LE8 score

	24-h movement pattern *z*-score	LE8 Score
	*R*-value, *P*-value	*R*-value, *P*-value
*Fasting*		
Glucose (mg/dl)^a^	– 0.328, *P* = 0.015	0.302, *P* = 0.099
Insulin (μU/ml)^a^	– 0.023, *P* = 0.869	– 0.130, *P* = 0.495
FFA (mEq/L)	0.004, *P* = 0.976	– 0.101, *P* = 0.602
CHOox (mg/kg/min)^b^	– 0.075, *P* = 0.597	– 0.362, *P* = 0.046
FOX (mg/kg/min)^b^	0.061, *P* = 0.666	0.477, *P* = 0.007
*Insulin-stimulated*		
Glucose (mg/dl)^b^	0.062, *P* = 0.655	0.223, *P* = 0.220
Insulin _Steady-State_ (μU/ml)	– 0.073, *P* = 0.609	– 0.287, *P* = 0.124
FFA _Steady-State_ (mEq/L)^b^	0.376, *P* = 0.007	– 0.155, *P* = 0.412
GIR (mg/kg/min)^b^	– 0.168, *P* = 0.224	0.135, *P* = 0.468
NOGD (mg/kg/min)^b^	– 0.192, *P* = 0.176	– 0.020, *P* = 0.917
CHOox (mg/kg/min)^b^	– 0.087, *P* = 0.543	– 0.197, *P* = 0.297
FOX (mg/kg/min)	0.093, *P* = 0.514	0.267, *P* = 0.183
FFA Suppression (%)^b^	– 0.289, *P* = 0.042	0.155, *P* = 0.423

*FFA* free fatty acids, *CHOox* carbohydrate oxidation, *FOX* fat oxidation, *GIR* glucose infusion rate, *NOGD* non-oxidative glucose disposal

^a^Data log-transformed for analysis

^b^Spearman correlation was used

### Mediation effect

The 24-h movement z-score was a significant mediator (*P* = 0.041) for fasting AIx75 on steady-state FFA with a mediation effect of 0.048. The total effect of fasting AIx75 on FFA was − 0.023, which was not statistically significant. This total effect was decomposed into a statistically significant mediation effect as a natural indirect effect (NIE) of 0.048 and a non-significant direct effect of − 0.071. The direct effect and the mediation effect had opposite signs, indicating the positive effect of movement *z*-score and the negative effect of fasting AIx75 on steady-state FFA. The 24-h movement z-score was also a significant mediator (*P* = 0.048) for fasting AP on steady-state FFA, with a mediation effect of 0.042. The total effect of fasting AP on steady-state FFA was 0.005, which was not statistically significant. This total effect was decomposed into a statistically significant mediation effect as a natural indirect effect (NIE) of 0.042 and a non-significant direct effect of − 0.037. The direct effect and the mediation effect had opposite signs, indicating the positive effect of movement *z*-score and the negative effect of the fasting AP on FFAs.

## Discussion

MVPA, SB, and sleep are the criteria for the 24-h movement behavior cycle [17, 18]. Some reports suggest that engagement in 2 to 3 of these behaviors reduces risk for obesity and metabolic syndrome [19, 20], while engagement in only 1 of the 3 behaviors is linked to higher CVD risk [21]. These behaviors are not mutually exclusive and have reciprocal relationships. For instance, people are often advised to replace SB time with more MVPA, and more MVPA may lead to additional sleep [22]. This complex multi-behavior lifestyle perspective has led to the adoption of analytic approaches to handle the colinear dependence of such outcomes on each other, including isotemporal substation modeling and compositional data analysis [23]. Findings from this literature suggest that reallocating time away from SB to MVPA and/or sleep induces multiple health benefits [24, 25]. However, a gap exists though in ease of use to the public. Herein, we show that a 24-h movement z-score aligning with recommendations (i.e., low z-scores) relates to cardiometabolic health. Individuals with aligned z-scores had favorable DBP, MAP, AIx75, AP, as well as elevated metabolic insulin sensitivity, NOGD, and insulin-stimulated FFA suppression compared to those with misaligned z-scores. This observation of FFA levels during insulin infusion was of particular interest since the 24-h movement z-score mediated the relationship between fasting AIx75 and AP with FFA during insulin stimulation. This suggests that movement may be a key mechanism linking arterial health and FFA metabolism. Moreover, we showed that while the individuals with aligned z-scores had favorable LE8 scores as would be expected, these two scores had different relationships with body composition, blood pressure regulation, and metabolic insulin sensitivity. Thus, these data highlight the potential utility of using the 24-h movement z-scores as an aggregate score of behaviors tied to health.

There has been debate in recent years as to whether physical activity/exercise can induce meaningful weight loss. Our findings suggest that, despite people having lower body weight when having favorable 24-h movement z-scores, no differences in body fat, visceral fat, or lean mass were observed. Only the LE8 correlated with our indices of body composition. While there may be a level of autocorrelation with the LE8 and body composition outcomes since BMI is a component of the LE8 calculation, it is also relevant that dietary pattern is a component within the LE8. Thus, we are cautious to infer that meeting 24-h movement pattern guidelines will not yield a healthy body composition as suggested by a recent meta-analysis [26] and observational study [27].

Insulin sensitivity as measured by fasting insulin and/or HOMA-IR has been related to 24-h movement patterns [27]. While these are fair clinical estimates, we show for the first time that people with obesity who have aligned 24-h movement z-scores tended to have higher GIR and NOGD than those with misaligned z-scores when measured via the clamp. Furthermore, these same individuals had increased insulin-stimulated FFA suppression. In contrast, the LE8 score showed no relationship with GIR, NOGD, or FFA suppression. However, it is worth noting that only LE8 scores correlated with fasting FOX, and this could suggest that other components of lifestyle behavior (e.g., diet) related to fuel selection. Together, these data suggest that aligning with 24-h movement pattern guidelines relates to better skeletal muscle and adipose tissue insulin sensitivity independent of other behaviors as well as HbA1c levels. Given there are no differences in fat mass between groups, this FFA finding is of particular interest as it could suggest adipose tissue function may be better. In fact, prior work shows that breaks in SB improve adipose tissue functionality [28]. Therefore, the present work confirms prior research suggesting that meeting 24-h guidelines can favor insulin sensitivity for reduced T2D and CVD risk.

We report that both aligned 24-h movement z-scores and LE8 scores related to lower blood pressure as well. It is important to recognize, though, that blood pressure is a component of the LE8 score calculation, potentially pointing to an autocorrelation. Importantly, only the 24-h movement *z*-score importantly related to AIx75. AIx75 has been considered an indirect measure of arterial stiffness [29]. AIx75 is determined from components of the pressure waveform, including the maximum systolic pressure minus pressure at the inflection endpoint (i.e., AP) and divided by total central pulse pressure. Herein, fasting AIx75 was lower in the aligned 24-h movement group, and the 24-h movement *z*-score, but not LE8, correlated with both fasting AIx75 and AP. This suggests that movement may independently influence fasting arterial waveforms. This aligns with prior work showing 2 weeks of caloric restriction had no effect on fasting AIx75, although it did lower post-prandial AIx75 in women with obesity [30]. Nonetheless, we observed no PWV differences between the groups. PWV is considered the gold standard for non-invasive assessment of arterial stiffness [31]. In turn, the observed AIx75 difference likely reflects altered aortic waveforms, possibly due to peripheral resistance, endothelial function, or sympathetic activity. In either case, alignment with 24-h movement pattern guidelines appears to favor reduced hypertension risk.

There are limitations to our study. Associations do not imply causation, and there is potential for autocorrelation of some outcomes potentially with the LE8. Future prospective trials assessing the impact of dynamic shifts in movement patterns on health outcomes are needed. Our results may not be generalizable since the population was mainly comprised of non-smoking women with obesity who were sedentary and without sleep disturbances. It is possible that our modest sample size of people with obesity limited our ability to adequately test some relationships or account for potential sex differences. Another consideration is that the misaligned 24-h movement pattern group had a higher weight than the aligned 24-h movement pattern group, which may be a confounder/explain other significant cardiometabolic relations. However, other measures, such as waist circumference, lean mass, and body fat, were not different between groups and minimize concern. Lastly, although the PSQI questionnaire is a validated approach against objective measures, this approach may over-/under-estimate sleep. Nevertheless, a strength of this study is that we used a z-score approach to assess the aggregate of movement patterns, which mirrors prior efforts in children [32] and adults as it relates to cardiometabolic disease [33].

In conclusion, individuals with obesity who align more with 24-h movement guidelines had more favorable metabolic insulin sensitivity, blood pressure, and aortic waveform findings than those who did not align with recommended movement patterns. However, only the LE8 score correlated with outcomes related to obesity. The collective findings showcase that the 24-h movement z-score can independently relate to cardiometabolic health. Further work is needed to understand the influence of 24-h movement patterns on cardiometabolic health to optimize strategies that prevent and/or delay lowering T2D and CVD risk.

## Data Availability

Upon reasonable request from the corresponding author.
